# Reliability and Repeatability of Pressure Center Analysis with Low-Dye Taping Using Force Platform Podiatry Sensors in Feet with Excessive Pronation

**DOI:** 10.3390/ijerph18063265

**Published:** 2021-03-22

**Authors:** Óscar Madruga-Armada, Ricardo Becerro-de-Bengoa-Vallejo, Marta Elena Losa-Iglesias, Cesar Calvo-Lobo, David Rodriguez-Sanz, Eva María Martínez-Jiménez, Victoria Mazoteras-Pardo, Marta San-Antolín

**Affiliations:** 1 Faculty of Health Sciences, Universidad Rey Juan Carlos, 28922 Madrid, Spain; madruga_oscar@yahoo.com (Ó.M.-A.); marta.losa@urjc.es (M.E.L.-I.); 2 Faculty of Nursing, Physical Therapy and Podiatry, Universidad Complutense de Madrid, 28040 Madrid, Spain; ribebeva@enf.ucm.es (R.B.-d.-B.-V.); cescalvo@ucm.es (C.C.-L.); evamam03@ucm.es (E.M.M.-J.); 3 Grupo de Investigación ENDOCU, Departamento de Enfermería, Facultad de Fisioterapia y Enfermería, Universidad de Casilla la Mancha, 45004 Toledo, Spain; victoria.mazoteras@uclm.es; 4 Department of Psychology, Universidad Europea de Madrid, Villaviciosa de Odón, 28670 Madrid, Spain; marta.sanantolin@universidadeuropea.es

**Keywords:** taping, reliability, analysis, foot

## Abstract

Background: The analysis of the center of pressure (COP) is a method used to assess the foot function, but its reliability and repeatability have not been evaluated. COP can be altered by diverse conditions, like an excessive foot pronation. Low-Dye taping is commonly used for the treatment of symptoms related to an excessive pronation. To date, no study has evaluated the effects of the Low-Dye taping on COP and the duration of its effects. Thus, the main purpose of this manuscript was to assess the reliability and repeatability of the percentage of center of pressure locus area (%CLA) in feet with an excessive pronation, and secondarily, to assess that the Low-Dye taping modifies the %CLA during the immediate 48 h. Methods: An observational study of the reliability and repeatability of the %CLA variable with the Low-Dye taping in feet with excessive pronation was carried out. We used the EPS-Platform to evaluate the results of the variable in 6 conditions in a first session to evaluate the reliability of the results. We compared the results of the first session with the results in a second session to evaluate the repeatability of the results. We also carried out an ANOVA test to evaluate the changes that the taping produced in the variable between without taping with the rest of the 6 conditions. Results: For the %CLA, we observed a reliability greater than 0.80, measured by the interclass ratio index, both in the first session before taping, and in the second session before taping, thus being a repeatability variable. In the following times, with taping, at 10 min with tape, at 20 min with tape, at 24 h with tape and at 48 h with tape; an interclass ratio coefficient (ICC) higher than 0.80 was again obtained, thus being a reliable variable in all measurements made. The Low-Dye taping did not change %CLA from the time the tape was put in until 48 h (*p*-value = 1.000). Conclusions: The %CLA variable, in feet with excessive pronation, proved to be a reliable variable in all the measurements obtained before putting on the tape and during the following 48 h with the tape, and a repeatable variable. The Low-Dye taping did not change the %CLA from the time the tape was put in until 48 h.

## 1. Introduction

Foot Pressure analysis (FPA) is a widely used tool for investigating foot posture and gait pattern [1,2,3,4,5]. Several parameters can be obtained by FPA. Among them, the center of pressure (COP) movement has been identified as a measure of neuromuscular control during posture and gait, and can be used to identify balance control, foot function, and treatment efficacy [6]. Because an increasing number of clinical decisions and treatment tools are made based on foot pressure analysis, it is important to know the reliability and repeatability of the values obtained with these devices [7].

One of the most common alterations of foot posture, that can induce passive instability and hypermobility of the foot joints, is overpronation [8,9]. Excessive pronation happens when the subtalar joint remains pronated beyond the middle phase of the gait cycle [10,11]. This overpronation can result in an increase in soft tissue stress and changes in the overall alignment of the lower limbs, often predisposing the affected person to injury to the lower limb [12].

Several surgical and nonsurgical methods have been proposed for treating symptomatic foot overpronation [13,14]. Among non-surgical treatments, the Low-Dye taping method is a taping treatment that was first described by Dr. Dye (1939) [15], which is commonly used by physiotherapists and podiatrists for the treatment of painful overpronation [16].

Therefore, we wonder if the percentage of the center of pressure locus area (%CLA) results are reliable and repeatable without taping and reliable with the use of the Low-Dye taping. We also wonder what immediate effects the Low-Dye taping will have on this variable and what will be the duration of these effects over time while the foot is taped. 

Thus, the main purpose of this manuscript was to assess whether %CLA is a reliable and repeatable variable in feet with an excessive pronation without any intervention and a reliable variable with the application of the Low-Dye taping. The secondary purpose of this manuscript was to assess that the Low-Dye taping modifies the %CLA variable during the immediate forty-eight hours after taping. 

## 2. Methods

### 2.1. Study Design

It was an observational study with a single-group repeated measures design. It was carried out by a single institution.

### 2.2. Ethical Considerations

The Research and Ethics Committee of Universidad Rey Juan Carlos, Spain, was the official entity that ruled the study, giving favorable authorization certificate. All volunteers gave written informed consent documentation before being part of this study. Human and ethical standards of experimentation were followed according to the Declaration of Helsinki and other organizations.

### 2.3. Subjects

The participants selected for the study were people who volunteered for it, from 1 April 2017 to 1 February 2019.

The recruitment was carried out through information leaflets.

Sampling was not randomized. It was a sample of consecutive non-probabilistic convenience, where the volunteers offered for the study were collected consecutively.

**Figure 1:**
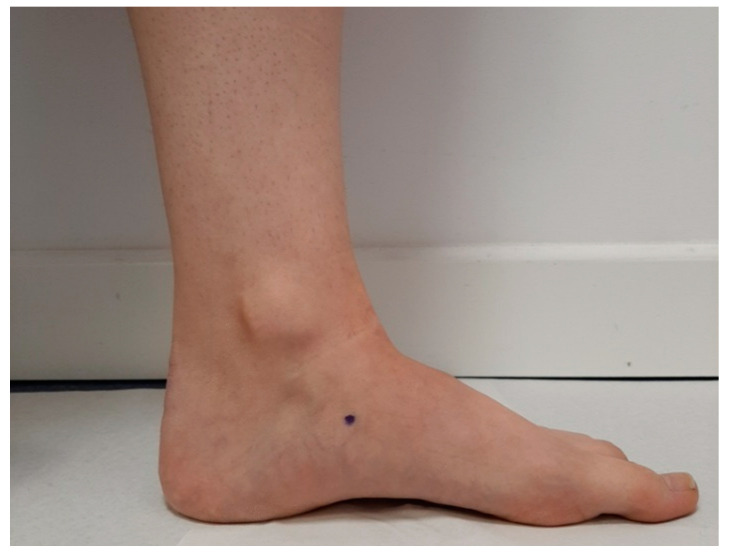
Signaling by a point of the most prominent aspect of the medial tuberosity of the navicular bone.

**Figure 2:**
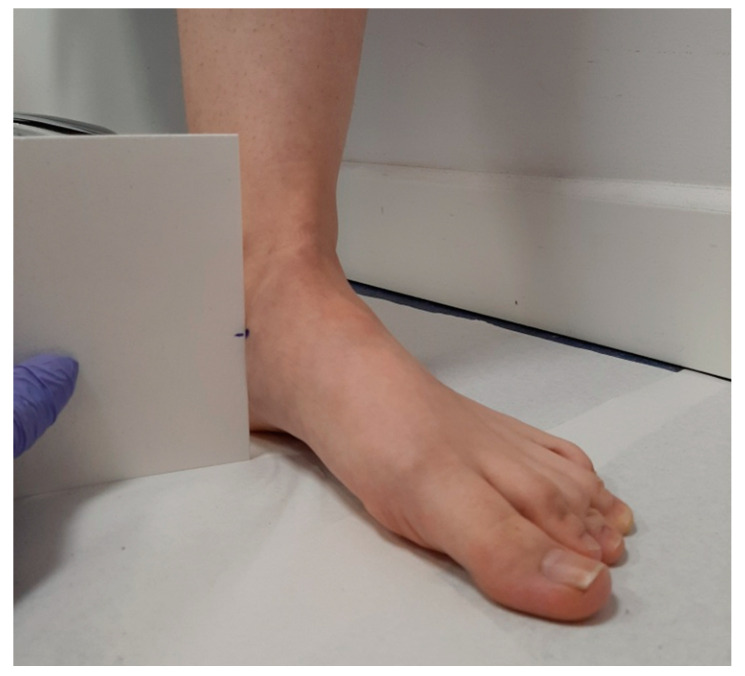
Measurement of the height point marked in the most prominent aspect of the medial tuberosity of the navicular bone with a neutral position of the foot.

**Figure 3:**
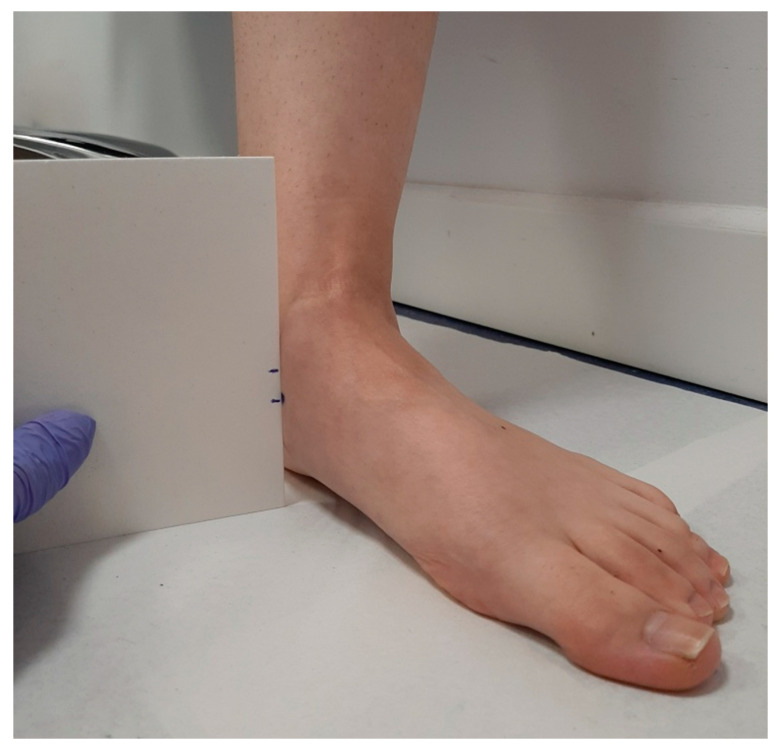
Measurement of the height point marked in the most prominent aspect of the medial tuberosity of the navicular bone with a relaxed position of the foot.

Regarding the criteria of inclusion and exclusion, a methodology similar to Nolan et al.’s study was followed [17]: 

Inclusion criteria for participation included willing and able to walk independently at a comfortable pace for two 10-min walking sessions and have a Navicular Drop test greater than 10 mm [17]. A Navicular Drop test greater than 10 mm was necessary for participation, as this is indicative of excessive pronation [18]. For the measurement of the Navicular Drop test, the technique described by Vinicombe et al. was used (2001) [19] (Figure 1, Figure 2 and Figure 3). 

Those with a Navicular Drop of 10 mm or less were excluded from the study [17]. The right foot was used for taping in subjects with bilateral excessive pronation [17]. The Navicular Drop test was measured in all participants by the principal researcher. 

Exclusion criteria for participation included an injury to the lower limb in the previous six months; gait affected by pain, injury, or neurological condition; a history of lower limb surgery; a known lower limb pathology, except excessive pronation; and the presence of any tape allergy sign, like excessive redness, rash, or skin peeling, after tape was removed [17].

Based on our inclusion and exclusion criteria, we included 35 participants in our study.

### 2.4. Taping Technique

**Figure 4:**
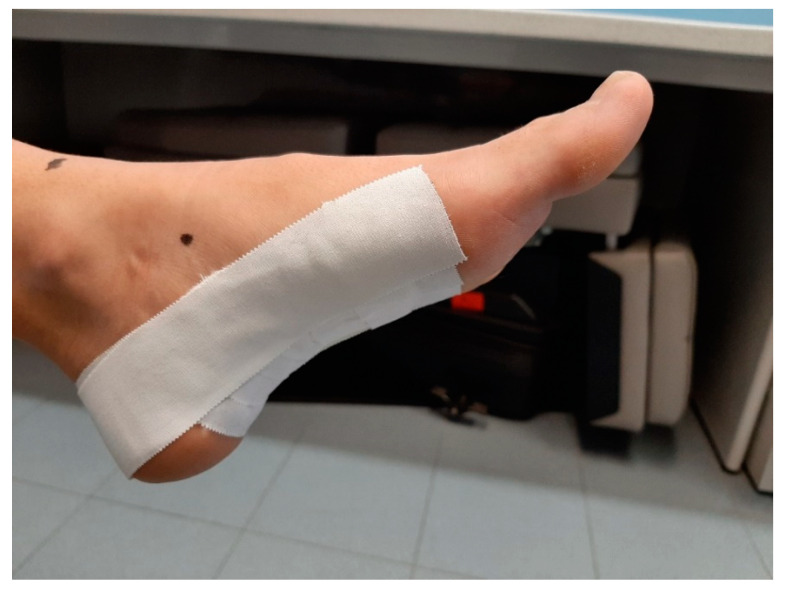
Low-Dye tape.

We used the standard Low-Dye taping technique, as it was described by Vicenzino et al. [20]. This is the gold standard technique in studies that use the Low-Dye taping [17,21]. Rigid Leukotape (38 mm) (Leukotape^®^ Sport, BSN Medical, Luxemburgo, Luxemburgo, www.bsnmedical.com (accesses on 16 March 2017) with a zinc oxide adhesive was applied while the subject was sitting with the foot in both talocrural and subtalar joint neutral position, as palpated by the investigator [17]. First the foot was patted down with a dry towel in order to maximize tape adherence [17]. To ensure consistency, the same investigator carried out all taping procedures involved in the study, following a standardized protocol [17]. The longitudinal arch support strips were placed in a side-to-medial direction starting at the head of the fifth metatarsal bone and ending at the head of the first metatarsal bone [20]. The strips for the transverse arch were then placed in a side-to-medial direction under the plantar surface of the foot starting on the anterior surface of the calcaneus bone and ending at the metatarsal heads [20]. The Low-Dye taping was completed with an additional strip for longitudinal arch support to provide additional assistance in holding the support strips of the transverse arch (Figure 4) [20]. Finally, to ensure that the Low-Dye tape adhered and to prevent it from being lost within 48 h, two strips of bandage were added on the back of the foot, thus finishing the taping [20,21].

The Low-Dye taping was made by the principal researcher in all subjects.

### 2.5. Instrumentation

**Figure 5:**
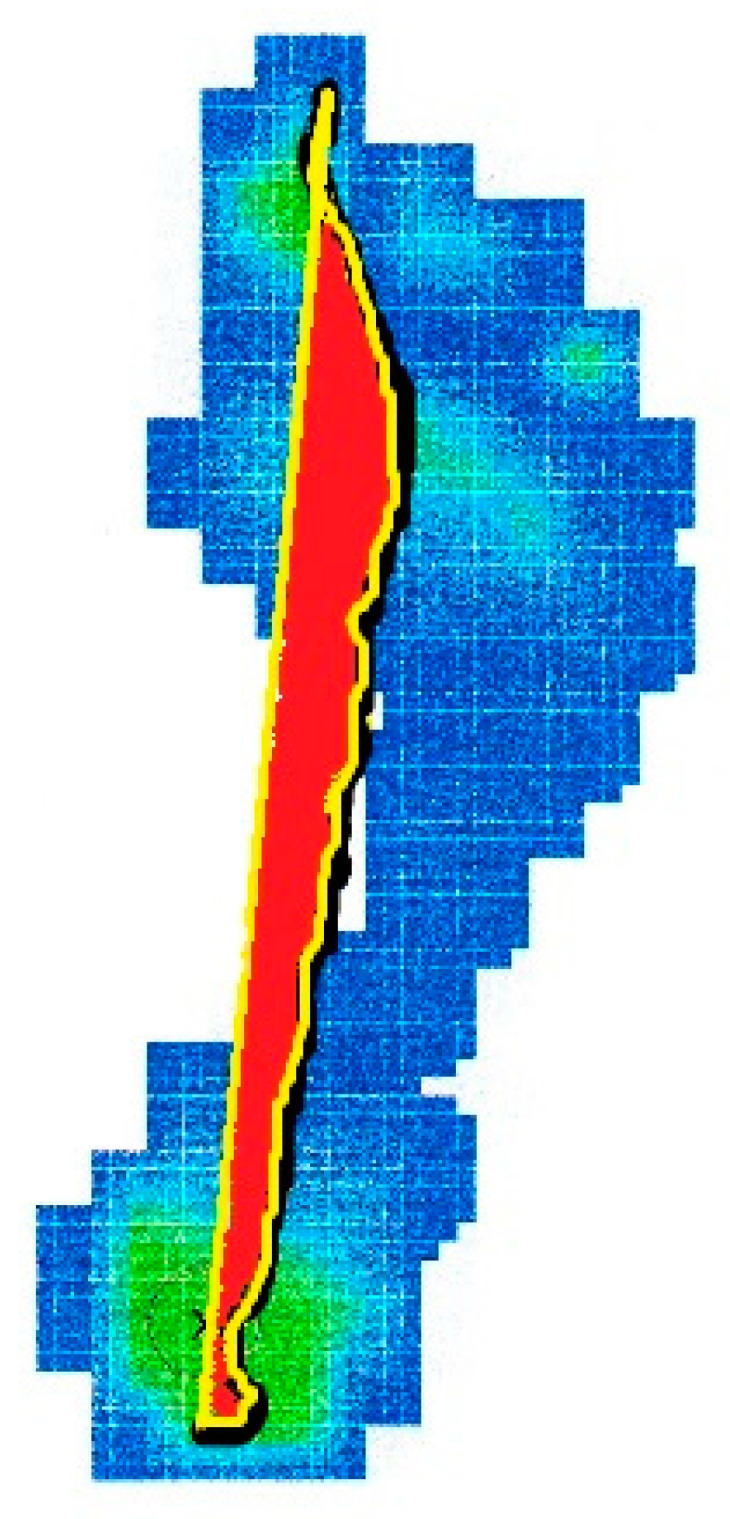
Percentage of the center of pressure locus area (red), defined like the ratio of the area encompassed by the center of pressure path (black, underlined above with yellow) and a line between the start and end points of the center of pressure path (straight yellow line) to the foot area.

The percentage of the center of pressure locus area (%CLA) is defined like the ratio of the area encompassed by the center of pressure path and a line between the start and end points of the center of pressure path to the foot area [22]. The %CLA, whose unit of measurement is pixel/mm^2^, was calculated using an image processing software (ImageJ; National Institutes of Health, Bethesda, Maryland, https://imagej.nih.gov/ij/) (accesses on 20 March 2017) (Figure 5) [22]. 

A portable digital pressure platform was used to gain the center of pressure path (EPS-Platform; Loran Engineering, Castel Maggiore, Bologna, Italy, www.loran-engineering.com) (accesses on 20 March 2017). The platform dimensions were 70 × 50 cm, the thickness was 5 mm, the weight was 7 kg, and the number of resistive sensors was 2304. Measurements were accurate to the nearest 0.001 kPa. The equipment met the CE Declaration of Conformity and was calibrated a few days before the study began. Vertical force was recorded at a frequency of 60 Hz. The platform was linked via an interface unit to a personal computer containing the data-collection software program Foot Checker, version 4.0 for Windows (Loran Engineering, www.loran-engineering.com) (accesses on 20 March 2017). The software produced pressure maps with pressure measured in kilopascals for each incident of data collection [23]. In a previous study, this platform has been shown to be reliable in clinic, with an ICC between 0.88 and 0.97 in all dynamic variables [24].

### 2.6. Data Collection

The data collected were the %CLA in dynamics using the pressure platform. 

A methodology similar to Nolan et al. [17] (2009) was followed to collect the data by adding two more conditions and using a pressure platform. 

Two data collection sessions were held. 

At the first session, six conditions were collected to evaluate the reliability of the results and the effects that Low-Dye taping generated on the %CLA variable: data after walking 10 m without tape, after walking 10 m with tape, after walk 10 min with tape, after walking 20 min with tape, after 24 h with tape and after 48 h with tape. After each condition, data were taken from the %CLA using the one-step technique. The 3-step data for the taped foot were taken after each condition.

The first condition was carried out without taping and %CLA data were taken [17]. After this condition, the investigator applied the Low-Dye tape to the participant’s foot [17]. Participants repeated the 10-m walk with the tape and %CLA data were taken [17]. Next, data were collected at 10 min and at 20 min of wearing the tape, while the subject was walking [17]. Because previous researchers [17,25] have evaluated the duration of the Low-Dye tape effects on plantar pressures for a short period of time, of 30 min of less, we considered it important to determine the duration of these effects over a longer period. Therefore, we extended the protocol proposed by Nolan et al. [17] to 24 and 48 h. Thus, after collecting the %CLA data the first day, during the first 20 min of activity, participants were quoted at 24 and 48 h to carry out data collection in both conditions. Participants were asked during these 24 and 48 h to carry out activities on their daily lives. Once the data were taken at 48 h, the tape was removed.

After a week, the participants were quoted again to repeat the baseline data collection without taping. This was the second session. We carried out this second session to compare the results obtained in the first session without taping, with the results obtained in this second session without taping, to assess the repeatability of the results. 

### 2.7. Study Variables

The study-dependent variable was the %CLA measured in pixel/mm^2^.

### 2.8. Sample Size Calculation

The sample size was calculated with software from the Unidad de Epidemiología Clínica y Bioestadística, Complexo Hospitalario Universitario de A Coruña (www.fisterra.com) (accesses on 1 March 2017), taking as reference a study in which the effects of the Low-Dye tape on peak plantar pressure were investigated immediately after its application [17]. At the beginning of the study, peak plantar pressure on medial forefoot were 199.83 ± 47.96 Kpa, and after completion of the study, the pressure in that area was 258.08 ± 98.56 Kpa [17]. With a 2-tailed test, a 95% confidence interval (α = 0.05) and with 80% statistical power (β = 20%), at least 35 participants were required in a single group.

### 2.9. Statistical Analysis

For statistical analysis of the data, mean and standard deviation (SD) were calculated with the confidence limit to 95% of the two sessions in which each test was performed.

The Kolmogorov–Smirnov test was performed to determine whether the variables follow a normal distribution (*p* > 0.05) and apply parametric test, or non-normal distribution (*p* < 0.05) to apply nonparametric test.

According to the Kolmogorov–Smirnov test, and considering that a *p*-value greater than 0.05 had a normal distribution, a normal distribution (*p*-value > 0.05) was obtained for the analyzed variable. Table 1 shows the results obtained after doing the normality tests of the studied variable.

Intrasession reliability and intersession reliability were evaluated together. Intrasession reliability consisted of describing the similarity of the measurements obtained in the analyses by repeating them three times, while the reliability of intersection (repeatability) is to describe the similarity between the measurements of the first session and the second session (in the second session, three analyses were performed 7 days after the first session).

Using the classification proposed by Landis and Koch, ICC values between 0.20 and 0.40 are considered to demonstrate reasonable reliability. Scores between 0.40 and 0.60 have moderate reliability, scores between 0.60 and 0.80 have considerable reliability, while in the highest category scores range from 0.80 to 1.00, which are considered almost perfect [26]. Other authors indicated that, to obtain reliability, an ICC value of at least 0.75 is required [27]. According to Portney and Watkins’ recommendations, clinical measurements with reliability coefficients greater than 0.90 improve the likelihood that the measurement will be valid [28].

For the absolute comparison of the results obtained in the two sessions, the coefficient of variation (CV) [28] was calculated, where the difference in means between sessions 1 and 2 is the standard deviation of the differences. The CV was used to refer to the relationship between the mean size and variability of each of the variables studied.

$$CV\%=\frac{DS}{media*100\%}$$

The match limit (LOA) [29] was calculated to define the amount of variation that may be influencing measurements. In the LOA, if the differences between measurements tend to match, the LOA result will close to zero.

The standard measurement error (SEM) was also calculated for each variable studied [29]. SEM is derived from the ICC and DS:

SEM=DS∗sqrt (1−ICC)

For its best interpretation, SEM was expressed as a percentage of the mean (SEM%) [29] as follows:

$$SEM\%=\frac{SEM}{average*100\%}$$

In addition, the minimum detectable change (MDC) was calculated, which is defined as the magnitude of the variation in the value of each scale below which that change can be interpreted as inherent in the variability of the valuation method itself, without a real change in the patient’s clinical situation. Statistical significance was accepted for *p*-values < 0.05. The MDC was calculated with a standardized mean (MDC 95%) [30] as follows:

$$MDC=1.96*SEM*sqrt^{2}$$

$$MDC\%=\frac{MDC}{mean*100\%}$$

The repeatability coefficient (CR) was calculated for intrasession analyses using the formula [31]:

CR=1.96∗DS of the difference in the compared data

The paired *t*-student parametric test was also used in cases of normality or Wilcoxon test in cases of non-normality, to determine systematic differences between the first and second session where the *p*-value is obtained, indicating that if *p* < 0.05, it is concluded that there is a difference between the two variables.

Normality values (VN) of the sample studied were defined for the area analyzed that were obtained from the formula:

VN=Mean ±1.96∗DS

From the VN result of each variable, its 95% range was calculated in the same way that the 95% CI was obtained for the ICC values of the variables, as explained above.

The values obtained in the validation will also be represented graphically with the Bland–Altman method [29]. This procedure evaluates the concordance between the two sessions by graphically representing the difference between each pair of values (order axis) versus the mean of each pair of values (abscissa axis).

The independent *t*-student test was used to compare demographic variables like age, weight, height, and body mass index (BMI), based on the gender of the participants.

An ANOVA test for repeated measures was used to compare the results of the %CLA without the tape with the rest of the conditions, to see if there were differences when the tape was placed on the subject.

Statistical analysis was performed using SPSS 17.0 for Windows (SPSS, Inc., Chicago, IL, USA). It was considered statistically significant with a *p*-value < 0.05 and with a confidence interval of 95%.

## 3. Results

The results presented in the following section are based on the evaluation of 35 healthy participants with a Navicular drop test greater than 10 mm. 

Table 2 shows the sociodemographic characteristics of the participants. Results were expressed by their mean and standard deviation with a 95% confidence interval (95% CI). 

It was observed that there were no significant differences (*p*-value > 0.05) for the sociodemographic variables studied.

To verify the reliability of the variable studied at the intraobserver level in a quantitative way, the interclass correlation coefficient (ICC) was calculated, and the classification proposed by Landis and Koch [26] was used, where an ICC between 0.20 and 0.40 is considered as a reasonable reliability, scores between 0.40 and 0.80 as considerable reliability and scores between 0.80 and 1.00 as near perfect reliability.

Table 3 shows the reliability of the variable studied before applying the taping, both in the first session and in the second session. At the first session, an interclass ratio coefficient (ICC) of 95% = 0.818 (0.708–0.896) was observed. In the second session, an ICC of 95% = 0.934 (0.897–0.961) was observed. 

Table 4 shows the reliability results of the variable studied during the intersession without taping (the repeatability of the results), obtaining a *p*-value = 0.747 and an ICC (95% CI) = 0.939 (0.878–0.968).

Then we proceed to assess the reliability of the variables studied in a qualitative way by graphically representing them using the Bland–Altman method, comparing the results obtained in the first session and in the second session without taping.

**Figure 6:**
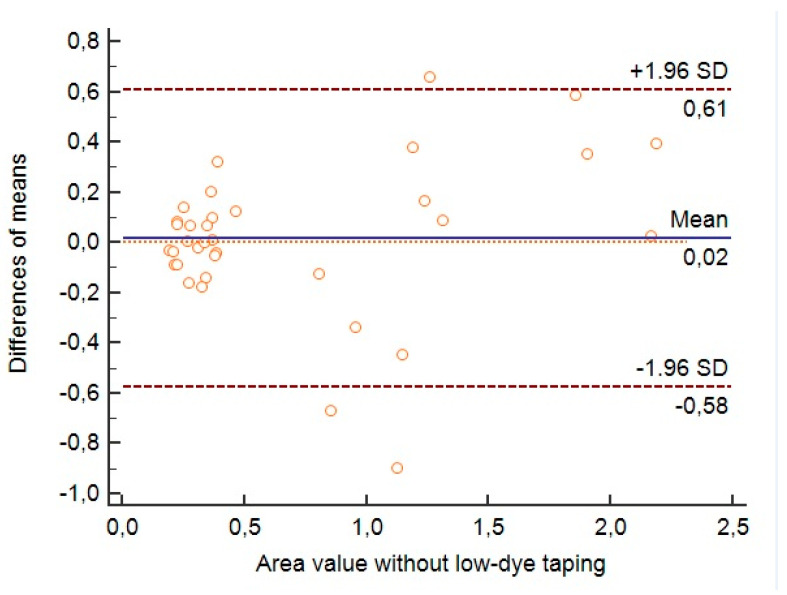
Bland–Altman graph comparing the results obtained for the variable Percentage of the Center of Pressure Locus Area without taping in the first session and in the second session of the participants. Abbreviations: Mean, media; SD, standard deviation.

In Figure 6, using the Bland–Altman graph, the dispersion of the results obtained in the first session and in the second session without tape for the variable %CLA is expressed qualitatively. It was observed a little dispersion of the results, noting that most of them are close to the mean, except for 3 measurements that are established outside the 95% confidence interval.

Once the reliability was assessed quantitatively and qualitatively in the first and second sessions, as well as the repeatability in the intersession, the reliability and normality values were assessed in the same variable throughout the time in which the participant wears the tape during the first session.

Table 5 shows the reliability results obtained at different times with the taping on, observing an ICC > 0.80 for all measurements.

Finally, we evaluated the effects of the Low-Dye taping on the %CLA variable, performing the ANOVA test for repeated measures on the variables studied, comparing the first time, without taping, to the other times, in the first session. When comparing the first time, without tape, to the other times, it was observed that there were no significant differences, with a *p*-value > 0.05 (Table 6).

## 4. Discussion

This study investigated the reliability and repeatability of the percentage of the center of pressure locus area (%CLA) in feet with an excess of pronation defined by a Navicular Drop test greater than 10 mm. We also evaluated the effects of the Low-Dye taping on the %CLA immediately after its application, at 10 min, 20 min, 24 h, and 48 h; on feet with an excess pronation defined by a Navicular Drop test greater than 10 mm.

Our main objective was to evaluate the reliability and repeatability of the variable studied. For the %CLA, we observed a reliability greater than 0.80, measured by the interclass ratio index, both in the first and second sessions before taping. The following times in the first session, with foot immediately after taping, at 10 min taping, at 20 min taping, at 24 h taping and at 48 h taping; an ICC higher than 0.80 was again obtained. 

This variable was originally mentioned for the first time in the article by Sugawara et al. [22], who assessed the quantitative distribution of pressures in patients with anterior cruciate ligament injury while walking using parameters calculated based on the center of pressure, including the variable of our study, the %CLA. They observed a shortening of the COP trace in the nondominant-side ACL-deficient group. In this study by Sugawara et al. [22], the reliability and repeatability of the variables used was not evaluated; therefore, our study was the first to evaluate the reliability and repeatability of the pressure center area as a variable.

Only one previous study has analyzed the reliability and repeatability of different dynamic variables using the pressure platform used in our study [23]. In the study by Becerro de Bengoa Vallejo et al. [23], they obtained a reliability measured by the ICC of 0.706 to 0.972 for the dynamic variables analyzed: mean pressure, integral pressure time, contact time or duration, peak pressure or maximum and integral pressure force time or force. These results are like those obtained in our study for the variable %CLA, which was shown as a reliable and repeatable dynamic variable.

Until now, according to our literature search, different studies have evaluated the effects of Low-Dye taping on plantar pressures [17,32,33,34,35], although the reliability and repeatability of the variables studied have not been evaluated. Only in the studies by Russo et al. [34] and Newell et al. [35] was the reliability of the pressure platform used in each one of them using the peak pressure variable evaluated, but they used measurement systems using different pressure platforms from each other and different from ours.

The other objective of this manuscript was to evaluate the effects of the Low-Dye taping on the %CLA variable. We hypothesized that these effects will not last over time, seeing a progressive loss of the effects on the %CLA until a situation like before taping. 

In our study, we observed that the application of this taping technique did not produce any increase or decrease effect on the %CLA variable. 

In our bibliographic search, only 5 studies [17,32,33,34,35] evaluated the effects of Low-Dye taping on plantar pressures in healthy subjects, which only 4 used subjects with a positive Navicular Drop test [17,32,34,35] compatible with excessive pronation, as in our study. In these studies, only the effects of the Low-Dye taping on the peak of plantar pressures are evaluated [17,32,33,34,35] or mean plantar pressure [32]. Lange et al. (2004) [32] observed a decrease in plantar pressures under the heel and forefoot, while a significant increase in pressures under the lateral region of the midfoot and toes. Russo et al. (2001) [33] also observed a decrease in plantar pressures in the medial midfoot and a significant increase in pressures under lateral midfoot. O’Sullivan et al. (2008) [34] obtained a significant increase in the plantar pressures in the lateral region of the midfoot and a significant decrease in the rearfoot and medial region of the forefoot. These changes suggest a decrease in foot pronation [32,33,34].

However, according to our review, no previous study has evaluated the effects of Low-Dye taping on other dynamic variables, like the center of pressure variables, so it is not possible to compare our results with previous studies.

Nolan et al. [17] was the first who evaluated the duration of the effects of the Low-Dye taping on plantar pressures over time, evaluating the immediate effects on plantar pressures and the duration of these effects after 20 min of physical activity. They observed that at 10 min of taping, the plantar pressure values in the lateral region of the forefoot returned to values like those prior to the placement of the Low-Dye tape [17]. No study has evaluated the duration of the effects of the Low-Dye taping on plantar pressure variables after 30 min of activity, because previous research suggests that Low-Dye tape completely loses its effect on foot movement after 30 min of gait [25]. Due to previous researchers [17,25] having evaluated the duration of the Low-Dye tape effects for a short period of time, of 30 min of less, we consider it important to determine the duration of these effects over a longer period.

In our study, we observed that the application of the Low-Dye taping technique did not produce any increased or decreased effect on %CLA, so we were not able to determine the duration of the taping effects in a time greater than 30 min.

As limitations in our study, the size of the foot of each subject was not considered, so the number of strips used to perform the taping varied by subject, making it impossible to perform an identical taping between subjects, although to homogenize this situation, the tape was applied with the maximum possible tension and was always performed by the same researcher. Similarly, the weight of the participants and the possible effect it could have on the effects of the Low-Dye taping were not considered. We believe that the subject’s weight is a factor that can influence the size of the Low-Dye taping effect, as well as the duration of the effect. Another limitation that we observed in our study is that the participants were not used to walking barefoot on the pressure platform, although the procedure was performed in this way to avoid the influence of footwear on the taking of the variables under study. There was no control group where a sham taping was placed as a placebo to compare the results. There was no comparison between %CLA and other validated measurements, parameter of tools. Finally, as a limitation, the subjects were not randomly selected, but it was a consecutive non-probabilistic convenience sample.

So far, this study is the first to evaluate the reliability and repeatability of the %CLA variable, as well as the first study to evaluate the effects of the Low-Dye taping on the center of pressure. Future studies should analyze the importance of this variable in other foot morphotypes and in various sorts of pathologies related to the lower limb. Future studies should compare this measurement with other validated measurements, also. Future studies should assess the reproducibility of this variable as well.

## 5. Conclusions

The variable percentage of the center of pressure locus area, in feet with excess pronation, proved to be a reliable and repeatable variable. The variable percentage of the center of pressure locus area proved to be a reliable variable in all the measurements obtained during the following 48 h with the tape on. 

The Low-Dye taping did not generate a decrease or increase in the variable percentage of the center of pressure locus area. The effects of the Low-Dye taping on the percentage of the center of pressure locus area were not observed to last 10 min, 20 min, 24 h, and 48 h after wearing the taping.

## Figures and Tables

**Table 1:** Normality test of the variable percentage of the area of the pressure center in the population in the first session, second session and intersession.

	First Session		Second Session		Intersession	
	Shapiro–Wilk	Kolmogorov–Smirnov	Shapiro–Wilk	Kolmogorov–Smirnov	Shapiro–Wilk	Kolmogorov–Smirnov
	*p*-Value	*p*-Value	*p*-Value	*p*-Value	*p*-Value	*p*-Value
%CLA	0.762	0.255	0.804	0.317	0.779	0.301

Abbreviations: %CLA, Percentage of Center of Pressure Locus Area. 95% CI, 95% Confidence Interval. Statistical significance for a *p*-value < 0.05, with a 95% confidence interval.

**Table 2:** Sociodemographic characteristics of the study participants.

	Men (*n* = 10) Mean ± SD (IC 95%)	Women (*n* = 25) Mean ± SD (IC 95%)	Total (*n* = 35) Mean ± SD (IC 95%)	*p*-Value
Age	22.80 ± 1.81 (21.67–23.92)	23.60 ± 6.82 (20.92–26.27)	23.37 ± 5.82 (21.44–25.30)	0.719
Height	71.80 ± 14.92 (62.55–81.04)	61.48 ± 14.36 (55.84–67.11)	64.42 ± 15.06 (59.43–69.42)	0.066
Weight	174.10 ± 11.47 (166.98–181.21)	167.20 ± 8.25 (163.96–170.43)	169.17 ± 9.64 (165.97–172.36)	0.054
BMI (kg/m^2^)	23.62 ± 3.85 (21.23–26.01)	21.90 ± 4.14 (20.27–23.52)	22.39 ± 4.08 (21.04–23.74)	0.265

Abbreviations: DS, Standard deviation; kg, Kilogram; cm, centimeters; BMI, Body Mass Index; 95% CI, 95% Confidence Interval; Statistical significance for a *p*-value < 0.05, with a 95% confidence interval. *p*-value calculated using the independent Student *t*-test.

**Table 3:** Reliability of the variable percentage of the pressure center area without taping in the first and second sessions.

	Mean ± SD (IC 95%)	CV (%)	ICC (IC 95%)	SEM	%Error SEM	MDC	VN (VN Inf–VN Sup)
%CLA first session (pixel/mm^2^)	0.71 ± 0.02 (0.70–0.89)	3.310	0.818 (0.708–0.896)	0.010	1.412	0.028	0.714 ± 0.045 (0.667–0.760)
%CLA second session (pixel/mm^2^)	0.72 ± 0.02 (0.89–0.96)	3.489	0.934 (0.897–0.961)	0.006	0.896	0.017	0.721 ± 0.039 (0.672–0.770)

Abbreviations: %CLA, Percentage of Center of Pressure Locus Area. SD, standard deviation; CV, coefficient of variation; ICC, interclass ratio coefficient; 95% CI, 95% confidence interval; SEM, standard measurement error, MDC, minimal detectable change; VN, normal values; Inf, lower; Sup, superior. Statistical significance for a *p*-value < 0.05, with a 95% confidence interval.

**Table 4:** Reliability of the percentage of the center of pressure locus area in the intersession without taping.

	**First Session**	**Second Session**	**Intersession**	***p*-Value**	**ICC** **(IC 95%)**	**CV (%)**
%CLA (pixel/mm^2^)	0.71 ± 0.02 (0.70–0.89)	0.72 ± 0.02 (0.89–0.96)	0.70 ± 0.01 (0.87–0.96)	0.747	0.939 (0.878–0.968)	1.665
	**SEM**	**%Error SEM**	**MDC**	**CR**	**LoA (IC95%)** **(LoA Inf–LoA Sup)**	**VN** **(VN Inf–VN Sup)**
%CLA (pixel/mm^2^)	0.002	0.411	0.008	0.593	0.0166 (−0.576–0.609)	0.705 ± 0.021 (0.682–0.728)

Abbreviations: %CLA Percentage of Center of Pressure Locus Area. SD, standard deviation; CV, coefficient of variation; CHF, interclass ratio coefficient, 95% CI, 95% confidence interval; SEM, standard measurement error, MDC, minimal detectable change; CR, repeatability coefficient; LOA, VN concordance limit, normality values; Inf, lower; Sup, superior. Statistical significance for a *p*-value < 0.05, with a 95% confidence interval.

**Table 5:** Reliability of the percentage of the pressure center in the different times with the taping.

	Mean ± SD (IC 95%)	CV (%)	ICC (IC 95%)	SEM	%Error SEM	MDC	VN (VN Inf–VN Sup)
%CLA low-dye tape (pixel/mm^2^)	0.71 ± 0.05 (0.72–0.90)	8.154	0.829 (0.724–0.902)	0.024	3.372	0.066	0.710 ± 0.098 (0.597–0.824)
%CLA low-dye tape 10 min (pixel/mm^2^)	0.76 ± 0.02 (0.69–0.89)	3.644	0.813 (0.699–0.892)	0.012	1.575	0.033	0.765 ± 0.039 (0.710–0.819)
%CLA low-dye tape 20 min (pixel/mm^2^)	0.73 ± 0.02 (0.78–0.92)	2.768	0.87 (0.785–0.926)	0.007	0.998	0.020	0.736 ± 0.039 (0.696–0.776)
%CLA low-dye tape 24 h (pixel/mm^2^)	0.70 ± 0.03 (0.80–0.93)	4.907	0.883 (0.807–0.934)	0.011	1.678	0.032	0.700 ± 0.058 (0.633–0.768)
%CLA low-dye tape 48 h (pixel/mm^2^)	0.70 ± 0.03 (0.82–0.94)	5.593	0.894 (0.823–0.940)	0.012	1.821	0.035	0.701 ± 0.058 (0.624–0.777)

Abbreviations: %CLA, Percentage of Center of Pressure Locus Area. 10 min, 10 min; 20 min, 20 min; 24 h, 24 h; 48 h, 48 h. SD, standard deviation; CV, coefficient of variation; ICC, interclass ratio coefficient; 95% CI, 95% confidence interval; SEM, standard measurement error, MDC, minimal detectable change; VN, normal values; Inf, lower; Sup, superior. Statistical significance for a *p*-value < 0.05, with a 95% confidence interval.

**Table 6:** Statistical significance comparing results without taping with results over time in the variable studied.

Variable	*p*-Value without Taping VS. Low-Dye Tape	*p* without Taping VS. Low-Dye Tape 10 min	*p* without Taping VS. Low-Dye Tape 20 min	*p* without Taping VS. Low-Dye Tape 24 h	*p* without Taping VS. Low-Dye Tape 48 h
%CLA (pixel/mm^2^)	1.000	1.000	1.000	1.000	1.000

Abbreviations: %CLA, Percentage of Center of Pressure Locus Area. Statistical significance for a *p*-value < 0.05, with a 95% confidence interval.

## Data Availability

Not applicable.
